# Estimating the Total Societal Cost of a Hexavalent Vaccine versus a Pentavalent Vaccine with Hepatitis B in South Korea

**DOI:** 10.3390/vaccines11050984

**Published:** 2023-05-15

**Authors:** Serim Min, Sun-Hong Kwon, Yeon-Woo Lee, Jung-Min Lee, Eun Jin Bae, Eui-Kyung Lee

**Affiliations:** 1 School of Pharmacy, Sungkyunkwan University, Suwon 16419, Republic of Koreash.kwon@g.skku.edu (S.-H.K.);; 2 Sanofi Korea Co., Ltd., Seoul 06578, Republic of Korea

**Keywords:** cost minimization, hexavalent, National Immunization Program, vaccine

## Abstract

In South Korea, the ready-to-use hexavalent vaccine (against diphtheria, tetanus, pertussis, poliovirus, Haemophilus influenzae type b, and hepatitis B) is not listed despite its facility of no need to reconstitute. It, therefore, has the potential to augment the efficiency of prevention against the six infectious diseases, and it may reduce vaccine-related errors of reconstitution when compared with the currently used vaccination scheme of the pentavalent vaccine with the additional shots against hepatitis B. Given the assumed clinical equivalence between the two vaccination schemes, a cost-minimization analysis has been performed from a societal perspective including all the medical and non-medical direct and indirect costs when vaccinating one birth cohort. The results indicate that the ready-to-use hexavalent vaccine induces a cost reduction of KRW 47,155 (USD36.22) per infant or 12,026 million Korean Won ($9,236,417) in total for the whole birth cohort with 260,500 children. Using the ready-to-use hexavalent vaccine causes a lower infection rate, has fewer vaccination sessions, and may save much time as compared with the current vaccination scheme in place. The ready-to-use hexavalent vaccine may, therefore, benefit the National Immunization Program by reducing the total societal costs of vaccination while improving convenience of infants, parents, and medical care professionals.

## 1. Introduction

Combination vaccines consist of bringing together particles comparable to single-component vaccines that are effective in the prevention of multiple infectious agents causing disease [1]. Administering multiple vaccines in a single shot has many advantages like streamlining timetables [2], fewer injections, little distress of the infant, higher rates of conformity with dense vaccination schedules [3], better vaccine coverage, appropriate time of shots [4], lower injection costs, more affordable space to store products, and lower rates of immunization errors [1,5]. According to the Advisory Committee on Immunization Practices (ACIP), the American Academy of Pediatrics, and the American Academy of Family Physicians, these strengths render combination vaccines to be much more preferred than having inoculations split into equivalent component vaccines [6]. Moreover, those split vaccines have received negative feedback from suppliers and recipients, and they are as well as are associated with risks for side effects [1]. Based on safety and immunogenicity data, the ACIP adopted, therefore, the use of the hexavalent vaccine in the federal Vaccines for Children program on 26 June 2019 [7].

South Korea may also have the option to use the hexavalent vaccine for infants, vaccinating the children at two, four, and six-months old in a three-dose primary series. However, this vaccine is currently not listed by the local health authorities. The hexavalent vaccine combines antigens against diphtheria, tetanus toxoids and acellular pertussis (DTaP), inactivated poliovirus (IPV), *Haemophilus influenzae* type b (Hib) conjugated to tetanus protein, and hepatitis B (HepB) (recombinant DNA) vaccine, termed as DTaP-IPV-Hib-HepB. Currently, three types of hexavalent vaccines are commercially available, namely, Infanrix-Hexa (GlaxoSmithKline, Victoria, Australia, 2000), Hexacima/Hexaxim/Hexyon (Sanofi Pasteur, Marcy L’Etoile, France, 2013), and Vaxelis (Sanofi Pasteur & Merck, Toronto, ON, Canada, 2016) [8,9,10,11].

One of them (Hexacima/Hexaxim/Hexyon) is a ready-to-use, fully liquid product that can be rapidly and safely administered skipping the reconstitution step as required for the pentavalent vaccines that are now registered and in use in South Korea [12,13]. With its ready-to-use status, this hexavalent vaccine type prevents several types of immunization errors that may happen [14]. A previous study has demonstrated that medical errors may occur most frequently during the reconstitution step [15]. Lee et al. (2021) investigated the number of vaccine-related errors in reconstituted and ready-to-use vaccines in a Korean clinical setting. Ready-to-use vaccines have reduced number of immunization errors and are preferred by healthcare providers [14]. In addition, the reconstitution step of the vaccine can lead to contamination with infections causing bacteraemia [16]. Larmené-Beld et al. (2019) stated that using the ready-to-administer prefilled sterilized syringes removed the preparation step (reconstitution) and reduced the adverse events associated with microbial contamination. The latter may incur additional costs [13]. A cost analysis of the hexavalent vaccine, with a focus on infection costs, is therefore warranted.

Also, a previous study identified the risk for infection in a real-life clinical setting during immunization processes and from claims data of a national database [13,17]. It revealed that the occurrence of infection following immunization may cause an additional cost burden. These findings were the incentives to investigate more in depth the rates of infection of pentavalent vaccines to be reconstituted versus the hexavalent vaccine that is ready to be used and estimate their cost difference. Studies on the economic value of the hexavalent vaccine suggested the following costs to be assessed: cost of the vaccine, cost of inpatient and/or outpatient visits, delivery cost, time cost, labor cost, cost for immunization errors and adverse events, and waste cost [18,19,20,21]. Our study adds the cost of infection contamination following immunization using the results of a model construct. 

Therefore, we aimed to evaluate the economic burden of the hexavalent and pentavalent vaccines with an additional shot of the HepB vaccine from a societal perspective. Furthermore, we believe this study will provide an improved approach to vaccine economic evaluation and allow the National Immunization Program (NIP) to introduce the hexavalent vaccine.

## 2. Materials and Methods

### 2.1. Vaccination Strategies

**Figure 1:**
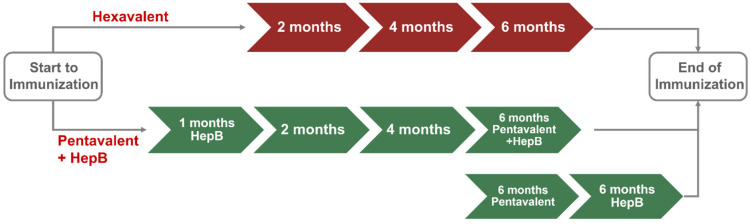
Summary of the 2 vaccine strategies.

The ready-to-use hexavalent vaccine (DTaP-IPV-Hib-HepB) is compared with the pentavalent vaccine (DTaP-IPV/Hib) with additional vaccine shots against HepB. The two vaccination strategies are summarized in Figure 1, that does not include the first vaccine shot against HepB at birth. Patients receiving the ready-to-use hexavalent vaccine visit the care unit after two, four, and six months after birth, resulting in a total of three medical care visits for vaccine administration. Patients receiving the pentavalent + HepB vaccines visit the medical care unit in the first, second, fourth, and sixth months after birth. At six months, the pentavalent + HepB vaccines can be administered simultaneously. If performed separately, it results in one additional fifth medical care visit.

### 2.2. Model Overview

**Figure 2:**
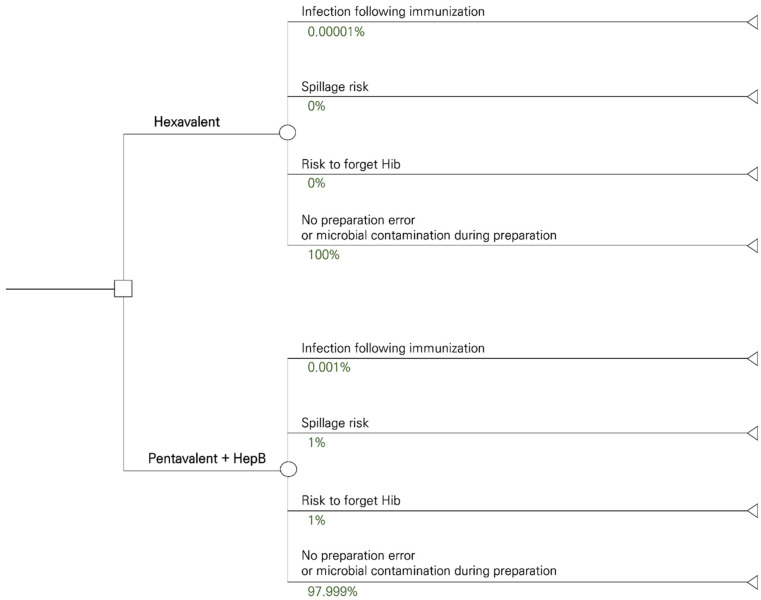
Decision tree model for vaccination (ready to use hexavalent versus pentavalent + HepB vaccines) with their adverse event risk assessment.

To compare the ready-to-use hexavalent vaccine with the pentavalent + HepB vaccines, a cost-minimization analysis is performed with a decision tree that is constructed to add probabilities of specific events occurring spontaneously and/or effectively [22]. The tree helps to determine the overall costs over a 6-month period of follow up for the individual (Figure 2). The infants received either the ready-to-use hexavalent vaccine or the pentavalent + HepB vaccine, to which per vaccination scheme is added to the frequency, expressed as probability measures of infection following immunization, spillage risk, risk of not getting Hib vaccine, or no preparation error or microbial contamination during preparation.

To make a full comparison of the cost differences between the two vaccination strategies, medical and non-medical costs are included. Medical costs consist of vaccine, administration, infection, and immunization error costs. Non-medical costs comprise transportation (direct) and time costs (indirect). Microsoft Excel is used to develop the decision tree model. The proxy good method is used to calculate the time cost included in the non-medical, indirect costs.

### 2.3. Input Parameters

For model construction, input parameters from multiple data sources are used and listed in Table 1.

#### 2.3.1. Vaccine-Related Probabilities

Data on inoculation completion rates and infection following immunization are obtained from the Korea Disease Control and Prevention Agency (KDCA). The rate of infection after immunization is calculated by dividing the number of infected patients (International Classification of Diseases-10th Revision (ICD-10) code: P36 Bacterial sepsis of newborns, A41 Other sepsis, T880 Infection following immunization) under the age of 15 years old by the total number of vaccinations. The online open database provided by HIRA is used to calculate the number of infected patients [6,23]. To estimate the infection rate of the ready-to-use hexavalent vaccine, a previous study helped to calculate the infection rate ratio as the infection rate in the pharmacy (0.08) divided by the infection rate in a clinical environment (7.47) [17]. It is assumed that at the pharmacy, the contamination risk is much lower compared with a clinical setting. The former could be a comparable situation to a ready-to-use condition of the hexavalent vaccine under study. The infection rate of the ready-to-use hexavalent vaccine is, therefore, calculated by multiplying the infection rate of the pentavalent vaccine with the infection rate ratio. The infection rates of the ready-to-use hexavalent and the pentavalent vaccines are therefore, respectively, 0.00001% and 0.001%. The probability of spillage risk and the risk of forgetting the Hib vaccine are obtained from the study by De Coster et al. (2015) [12].

#### 2.3.2. Costs

Medical and non-medical costs are included, the latter with time and transportation costs. The vaccine is administered to infants for which a caretaker needs to take time to go to a medical unit. This time of not being at work must be estimated and translated into monetary units when considering the societal perspective. The hexavalent vaccine cost has been assumed, as this vaccine is not included and listed in the present NIP. The pentavalent vaccine cost, HepB vaccine cost per dose, and the administration cost per dose are obtained from the cost lists for drugs and administration announced by the KDCA. Cost incurred by infection after vaccination is calculated using the HIRA data (ICD 10: P36, A41, and T880). Immunization error costs are calculated assuming that clinics use a new vaccine when an error occurred. The cost is, therefore, the same as the vaccine cost. Administration cost varies by type of vaccine and there is no cost when it is administered in a community health center. Accordingly, the administration cost also reflects the proportion of administrations done at a community health center (8%). Transportation costs, which is the round-trip cost of a single medical visit for getting the vaccine, are adopted from the outpatient medical costs presented in the 2005 Korea National Health and Nutrition Examination Survey, with the price index of 2020. For the time cost, proxy good methods referring to the hourly cost of infant care disclosed by the Ministry of Gender Equality and Family are applied for 2 h. All costs are presented in Korean Won (KRW, KRW 1302 = US 1 based on the exchange rate as of 16 March 2023).

### 2.4. Model Outcomes

The additional costs due to infection and errors during immunization are calculated using the decision tree model. The costs for each vaccination strategy are determined by summing the costs associated with the vaccination and the anticipated costs induced by visiting medical units under the national immunization program. Costs for 6 months are estimated when immunization is completed and not reported per inoculation event. Total costs per infant are calculated by summing the vaccine and administration costs, infection cost, immunization cost, transportation cost, and time cost. Subsequently, total cost and cost differences between the two strategies compared from a societal perspective are calculated by multiplying the total cost per infant with the number of infants in a birth cohort, based on the census 2021 from the Korean Statistical Information Service [24]. In addition, total costs and cost difference from the healthcare system perspective are also reported, which include vaccine, administration, and infection costs. Immunization error cost is not included in the healthcare perspective. The new vaccine cost is applied to the rest of the societal cost.

### 2.5. Probabilistic Simulation

To validate the model, a simulation exercise is developed setting some of the input parameters with probabilistic distributions, instead of fixed values. The selected parameters are the proportion of infants inoculated in the community health center, the proportion of infections following immunization, the infection rate ratio, and the probability of immunization error. In addition, the cost for infection, transportation, and time are also subject to probabilistic simulations. The attributes of each parameter with the different distributions, such as gamma, beta, or uniform distributions, that are applied are presented in Table 1. The type of distribution selected is determined based on the recommendation of selecting distributions for uncertain parameters in health economic evaluation [25]. A total of 1000 Monte-Carlo simulations are iterated to estimate the total costs of the ready-to-use hexavalent vaccines and of the pentavalent vaccine with HepB by changing the values of the input parameters that follow their distributions.

### 2.6. Sensitivity Analysis

One-way deterministic sensitivity analysis is also performed on key parameters to examine their robustness. Deterministic sensitivity analyses on the proportion of patients who are immunized in public health centers varied from 5 to 10%. As the ready-to-use hexavalent vaccine cost in Korea is not determined, the sum of the vaccine cost with the administration costs are varied from −5% to +5%. In addition, the risk of immunization infection varied from 0% to 2%, with 0.001% at the baseline. When estimating the time cost, the proxy good method applies a 2 h duration in the base-case analysis. Time costs are varied by applying a proxy method of 1 to 3 h. The proportion of infants who got the pentavalent and HepB vaccines together in one session at six months of age also varied from 0% to 100% in the sensitivity analysis.

## 3. Results

The cost-minimization analysis reveals that the ready-to-use hexavalent vaccine could be less costly with KRW 47,155 (US 36.22) in total per infant compared with the pentavalent vaccine or KRW 12,026 million (US 9.23 million) saved for the total birth cohort when considering the societal perspective (Table 2). The administration fee + the vaccine cost for the ready-to-use hexavalent vaccine is KRW 259,998 (US 199.69) per infant, which is higher than for the pentavalent vaccine + HepB. Including the immunization infection cost could result in a lower cost difference between the two vaccines from a health care perspective as this cost is higher for the pentavalent vaccines + HepB (KRW 8 ($0.006)) versus the ready-to-use hexavalent vaccine (KRW 0.084 (US 0.00006)). The ready-to-use hexavalent vaccine results in no extra cost for immunization errors, whereas the pentavalent vaccines with HepB incur a cost of KRW 2099 (US 1.61) per infant. Parents’ transportation costs for the ready-to-use hexavalent vaccine are KRW 12,128 ($9.32) lower than those for the pentavalent + HepB vaccine. Similarly, the ready-to-use hexavalent vaccine could save time cost compared with the pentavalent + HepB vaccines. Total costs are saved when the societal perspective is considered using the ready-to-use hexavalent vaccine, but it requires an additional cost of KRW 5489 (US 4.22) per infant when the perspective of the healthcare system is evaluated.

**Figure 3:**
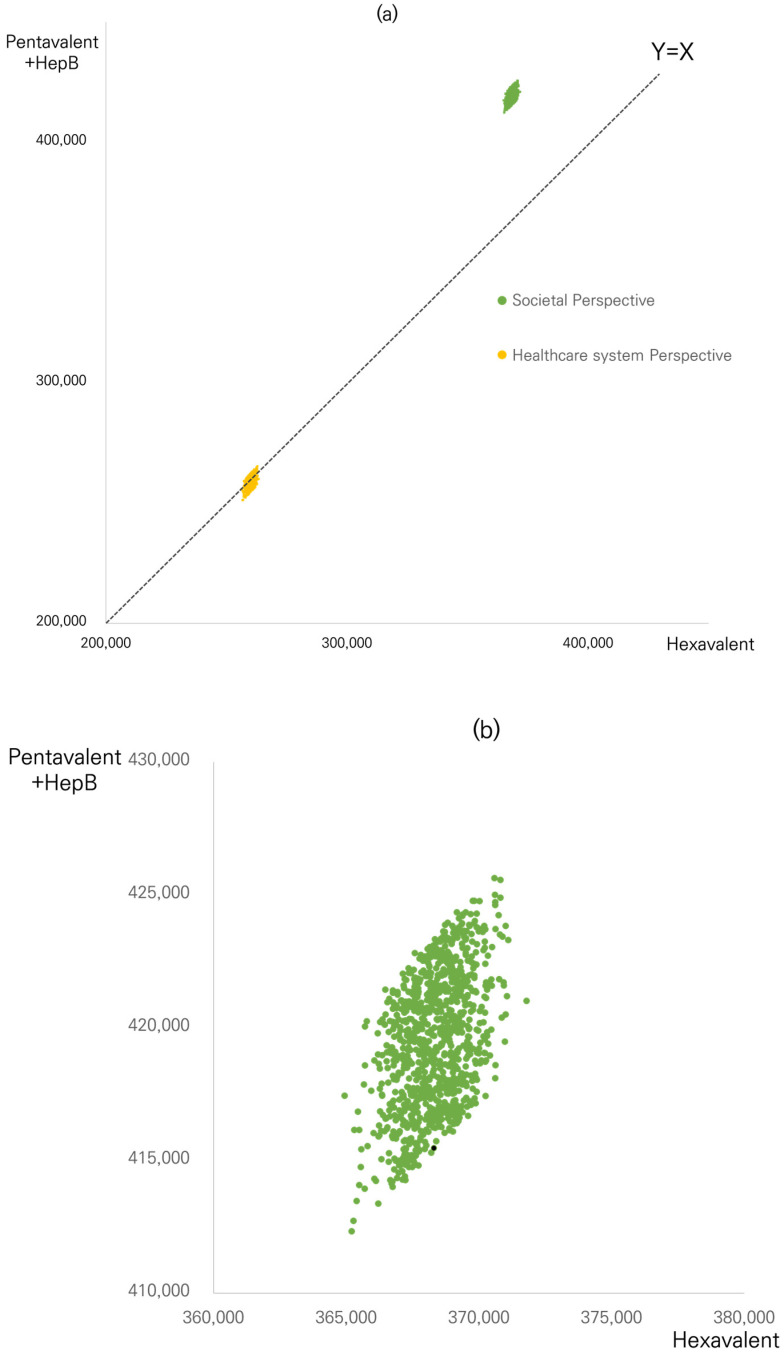

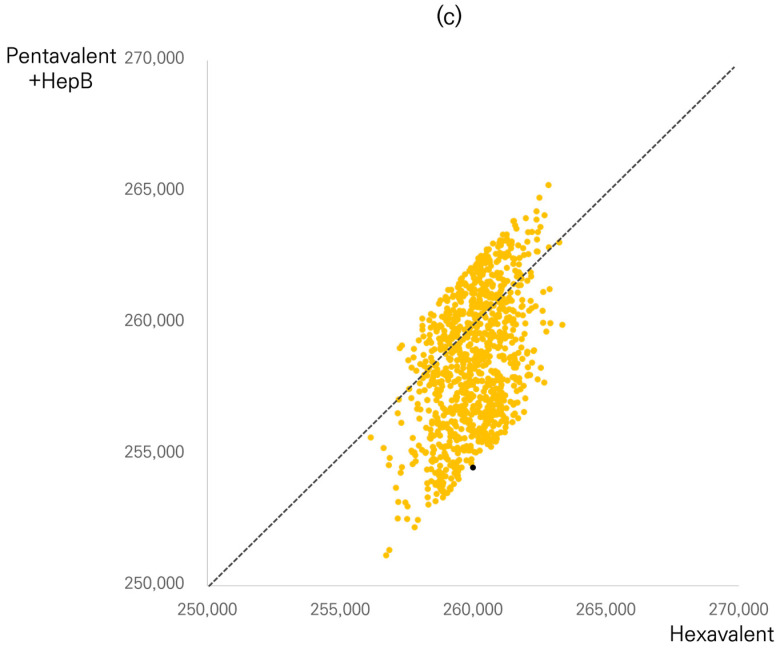
The total cost resulting from probabilistic simulation. (**a**) Total cost from the societal and healthcare system perspectives (KRW/infant). (**b**) Total cost from the societal perspective (KRW/infant). (**c**) Total cost from the healthcare system perspective (KRW/infant). Black dots in (**b**,**c**) indicate the base case of results. Hep B, Hepatitis B.

**Figure 4:**
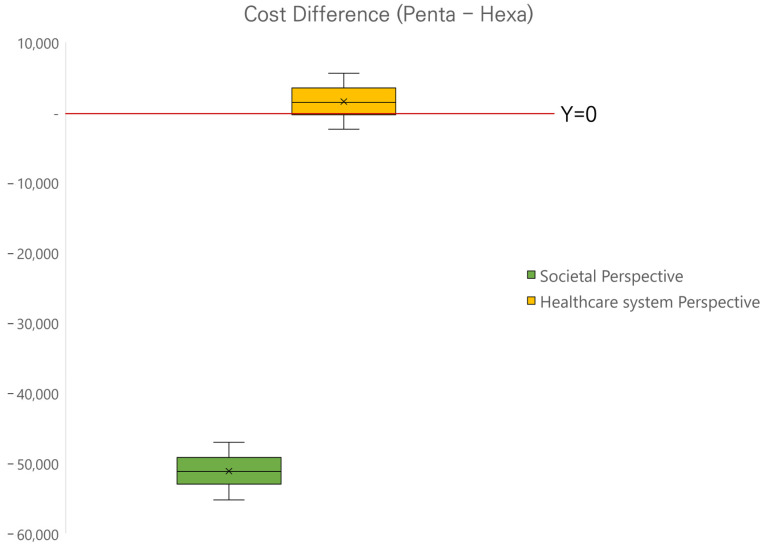
Total cost difference from probabilistic simulations (KRW).

Figure 3 shows the results of the probabilistic simulation as each point represents the total costs of using the hexavalent vaccine (X-axis) and the pentavalent vaccine (Y-axis) from the societal perspective (green dots) and the healthcare system perspective (yellow dots) when the input parameters are changed by each distribution. Based on 1000 model runs, all dots from the scatter plot of total costs from the societal perspective were above the line representing Y = X. Magnifying the scatter plots showed that the vaccination scheme with pentavalent vaccine plus HepB was higher than the hexavalent vaccine, indicating that the pentavalent vaccine was costlier (Figure 3b,c). However, for the scatter plots of total costs from the healthcare system perspective, 69.1% of the dots were under the line representing Y = X, implying that the total cost of the pentavalent vaccine was lower than that of the hexavalent vaccine, with a slight cost difference. In addition, we used Box plots to represent the quartiles of the cost difference of the pentavalent and the hexavalent vaccines from the results of probabilistic simulation for societal and healthcare system perspectives, respectively (Figure 4). The median difference between the pentavalent and the hexavalent vaccines from the healthcare system perspective was above zero, whereas the median difference from the societal perspective was less than KRW −50,000.

**Figure 5:**
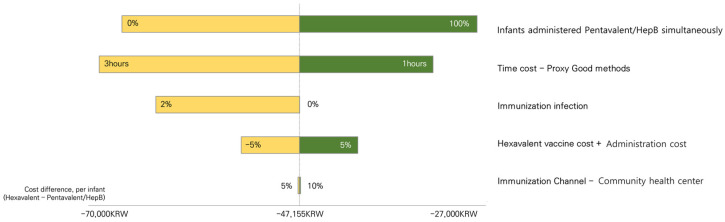
Results of sensitivity analysis. Tornado diagram assessing the impact of changes in variables. Yellow bars represent that the cost difference between the Hexavalent and Pentavalent + HepB is smaller, and green bars represent that cost difference is bigger than the base case (−47,155 KRW).

The results of the deterministic sensitivity analysis are shown in Figure 5. The total cost was reduced across all parameters with the hexavalent vaccine. The parameter that most affected the cost was the proportion of infants who were administered pentavalent and HepB simultaneously at six months. Cost is saved by using hexavalent from KRW 27,947 (US 25.13) to KRW 68,816 (US 52.85). Time cost estimation method also saved a large amount, which saved KRW 66,363 (US 50.97) per infant with the hexavalent vaccine. Other parameters affected the cost in the following order: the immunization infection rates; cost of the hexavalent vaccine and administration fee; and the proportion of visiting public health centers used for immunization.

## 4. Discussion

This cost-minimization analysis compares two vaccination strategies using the ready-to-use hexavalent vaccine with the pentavalent + HepB vaccines. From a societal perspective, the application of the ready-to-use hexavalent vaccine estimates a cost reduction of KRW 47,155 (US 36.22) per child vaccinated and KRW 12,026 million (US 9,236,417) for the whole birth cohort. Although the spending could be higher when only vaccine and administration costs are considered for the ready-to-use hexavalent vaccine as compared with the pentavalent + HepB vaccines, its overall costs could be lower in favor of the ready-to-use hexavalent vaccine because of reduced costs incurred by infections following immunization and reduced non-medical direct and indirect costs. The latter constitutes the most critical component of the total savings. Combination vaccines, that have different antigens in a single administration, have been promoted to overcome problems associated with multiple administrations of monovalent vaccines. The use of combination vaccines offers multiple benefits such as simplifying the administration process of the combination [2], decreasing the costs of combining and administering separate vaccines [1,5], and lowering the risk of delayed or missed vaccinations [12]. The present study confirms the cost savings obtained with a combination vaccine that is ready-to use due to fewer infections, fewer administration errors, and fewer visits. Even though local health authorities may pay more when bringing the ready-to-use hexavalent vaccine to the NIP, they may consider the importance of the other advantages that benefit infants and their parents. The probabilistic simulations that change input values of key parameters in this analysis, demonstrate that all simulations, conducted from a societal perspective, and are costlier for the pentavalent + HepB vaccines than the ready-to-use hexavalent vaccine (Figure 3a). When considering the healthcare perspective, 70% of the simulations show that the pentavalent vaccine could be cheaper, while 30% of simulations demonstrate results with a lower cost for the ready-to-use hexavalent vaccine.

An earlier study estimated the budget impact of introducing a fully liquid or ready-to-use hexavalent vaccine into the United Kingdom’s pediatric immunization program compared with another hexavalent vaccine requiring reconstitution. The study supported the advantages of fully liquid vaccines over reconstituted vaccines having the potential of cost savings by shortening vaccine preparation time and reducing the risk of vaccination error [19]. Similar results were seen when introducing the ready-to-use hexavalent vaccine into the national vaccination program in Peru that reduce total costs due to lower time costs despite an increase in vaccine costs [21]. A similar cost analysis was also performed for the DTaP-based hexavalent vaccine (DTaP-292 IPV-Hib-HepB) in Malaysia. The study analyzed the effects of replacing the pentavalent + HepB vaccines with the hexavalent vaccine. It analyzed the costs from a societal perspective. Reports revealed that the total cost was reduced, consistent with the results seen in this study [18,26].

Beyond the support from results of other studies, this study here exclusively includes costs resulting from infection after immunization. This infection cost is quite marginal compared with the overall cost of vaccination due to the low infection risk. However, one is able to lower the infection rate related to immunization that can be measured and is achievable with the introduction of alternative vaccines such as the ready-to-use vaccines. The HIRA statistics show that infection following any vaccination may occur in over 100 infants annually in South Korea. It leads to an additional cost of KRW 261,812 ($ 201) per infant. Therefore, including the cost of infection, when evaluating the economic burden of infant vaccines, is a necessity, as both administrations of the hexavalent and/or the pentavalent vaccines have risks for infection, especially when they need to be reconstituted. Meanwhile, there is a lack of critical information about infection rates following vaccination globally. To get infection rates included into our decision tree model, data from nationwide claims database are used provided by the HIRA. The HIRA contains claims data about approximately 50 million beneficiaries, covering all South Korean citizens. The results of this study should therefore be considered representative of the situation of the country.

This study has, like any other modeling study, limitations. The proxy good method is used to calculate the time cost with the hourly wage of the babysitter suggested by the Ministry of Gender Equality and Family that is assumed to be the right cost. Sensitivity analysis confirmed that robust results are obtained by conducting a cost analysis using the opportunity cost method or the proxy good method. To calculate the infection rate ratio of the ready-to-use hexavalent vaccine and compare that ratio with that of the pentavalent + HepB vaccines, contamination rates are used that were noted during medical preparation time in clinical versus pharmacy settings, reported by Larmené-Beld et al. (2019) [13]. However, in the case of the hexavalent vaccine that does not require reconstitution, as the vaccine is in a ready-to-use formulation, discrepancies are observed between the results of this study with previous studies. Meanwhile, because the ready-to-use formulation may have a lower contamination rate than that of pharmacies, the estimation of the ready-to-use hexavalent infection rate obtained using the value assumed in previous literature is considered a conservative assumption.

This study constructs a probabilistic decision tree model to include a range of realistic infection rates following immunization to be tested to compare costs when using the pentavalent + HepB vaccines or the ready-to-use hexavalent vaccine. Representative data from South Korea and nationwide statistics from claims data are taken to calculate the infection rate and its costs. In addition, time costs are considered to include the caregiver burden and showed improvements in convenience due to the decrease in the number of visits of children by their parents. Consequently, the inclusion of time costs reveals that the ready-to-use hexavalent vaccine could reduce much of the non-medical indirect costs, particularly the total cost of vaccination overall compared with the use of the current vaccine setting in Korea.

## 5. Conclusions

Introducing the ready-to-use DTaP-based hexavalent vaccine (DTaP-IPV-Hib-HepB) compared with the pentavalent + HepB vaccines may save total costs from a societal perspective while it could have a higher cost from a healthcare system perspective. However, the ready-to-use hexavalent vaccination scheme may have benefits in lowering the risk for infection, reducing the number of clinical visits, better handling, fewer administration errors, and limiting the number of injections. It may consequently lead to better acceptability of the vaccination program by parents and the medical corps, as it is more convenient by substantially reducing the socioeconomic burden.

## Figures and Tables

**Table 1:** Input parameters.

Parameter	Base-Case Value	Distribution for Probabilistic Simulation ^1^	Source
Infection following immunization			
Pentavalent	0.001% ^2^	Uniform (0.01–1%)	Base case: healthcare big data hub by HIRA (opendata.hira.or.kr) Range for probabilistic simulation: Larmené-Beld et al. (2019) [13]
Infection rate ratio	0.01 ^3^	Uniform (0.008–0.0155)	Larmené-Beld et al. (2019) [13]
Ready-to-use Hexavalent	0.00001% ^4^		Infection rate of pentavalent × infection rate ratio
Immunization error			
Spillage risk	1%	Beta	De Coster et al. (2015 [12])
Risk to forget Hib	1%	Beta	De Coster et al. (2015) [12]
Infants administered Pentavalent/HepB simultaneously at six months	60%		Assumed
Cost, KRW			
Vaccine cost per dose			
Ready-to-use Hexavalent	₩ 42,000	–	Assumed
Pentavalent	₩ 34,990	–	Unit cost announced by KDCA
HepB	₩ 3300	–	Unit cost announced by KDCA
Administration cost			
Community health center ^5^	₩ 0		
Clinic/hospital			
Ready-to-use Hexavalent	₩ 48,550	–	Assumed ^6^
Pentavalent	₩ 38,840	–	Unit cost announced by KDCA
HepB	₩ 19,420	–	Unit cost announced by KDCA
Infection cost per case	₩ 261,812	Gamma	Healthcare big data hub by HIRA (opendata.hira.or.kr)
Immunization error cost		Gamma	Assumed one additional vaccine dose
Ready-to-use Hexavalent	₩ 42,000		Same as a vaccine cost
Pentavalent	₩ 34,990		Same as a vaccine cost
HepB	₩ 3300		Same as a vaccine cost
Transportation cost per visit	₩ 8663	Gamma	2005 KNHANES report
Time cost per visit	₩ 27,440	–	KOSIS
Birth cohort (2021)	260,500	–	KOSIS
Inoculation completion rate	97.90%	–	Unit cost announced by KDCA

^1^ To estimate gamma and beta distributions, the mean and standard deviation is used by assuming the value of base-case and 10% of the mean. ^2^ The number of infected patients/total number of vaccinations (under 15) = 121/12,047,053 = 0.00001. ^3^ Infection rate in the pharmacy/infection rate in the clinical environment = 0.08 ÷ 7.47 = 0.01. ^4^ Infection rate of pentavalent × infection rate ratio = 0.00001 × 0.01 = 0.0000001. ^5^ Administration cost is at no cost when administered in community health center. Proportion of visiting community health center was 8% (National vaccination rate in 2020, reported by KDCA). ^6^ Calculated based on the rules of administration cost for a combination vaccine. KRW 1302 = US 1; KOSIS, Korean Statistical Information Service; KDCA, Korean Disease Control and Prevention Agency; HIRA, Health Insurance Review and Assessment Service; KNHANES, Korea National Health and Nutrition Examination Survey; CPI, Consumer Price Index; KRW, Korean Won.

**Table 2:** Base analysis results.

Variable	Hexavalent	Pentavalent + HepB	Difference (Hexavalent- Pentavalent + HepB)
Cost items per infant, KRW			
Vaccine cost + administration cost	₩ 259,998	₩ 254,501	₩ 5497
Infection cost following immunization	₩ 0.084	₩ 8	₩ −8
Immunization error cost	₩ 0	₩ 2099	₩ −2099
Transportation cost	₩ 25,989	₩ 38,117	₩ −12,128
Time cost	₩ 82,320	₩ 120,736	₩ −38,416
Total cost per infant, KRW			
Societal perspective	₩ 368,307	₩ 415,462	₩ −47,155
Healthcare system perspective	₩ 259,998	₩ 254,509	₩ 5489
Total cost of the birth cohort, ^1^ million KRW			
Societal perspective	₩ 93,929	₩ 105,955	₩ −12,026
Healthcare system perspective	₩ 66,307	₩ 64,907	₩ 1400

^1^ Total saving for a birth cohort (*n* = 260,500) reflected the inoculation completion rate (97.9%). HepB, HepatitisB.

## Data Availability

The data that support the findings of this study are available from the corresponding author upon reasonable request.
